# Supplementary material for: Evaluation of a Pilot Wellness Elective for Master of Public Health Students during the COVID-19 Pandemic
Source: Int J Environ Res Public Health. 2024 May 3;21(5):590. doi: 10.3390/ijerph21050590 (PMC11121606; doi:10.3390/ijerph21050590)

SUPPLEMENTARY

Table S1: “Culture of Wellness” course deliverables
Table S2: Pre-post survey given to students immediately before and immediately after the “Culture of Wellness” course
Table S3: Personal Health Improvement Plan topics chosen by students in the 2020 course
Table S4: Perceived Stress Scale pre-course survey results
Table S5: SMART goals for each of the 6 team projects
Figure S1: Perceived stress pre-course survey results in 2019 versus 2020
Figure S2: Five-month post-course survey question on importance of strategies to the students’ personal wellness
Figure S3: Five-month post-course survey on confidence in ability to apply the strategies
Figure S4: Five-month post-course survey question on frequency of application of strategies

Table S1: “Culture of Wellness” course deliverables

Deliverable	Description
Pre-class readings and reflections	Weekly written reflection provided an opportunity to display understanding and personal insight of course readings. Students were given a prompt to respond to with an expected paper length of 200-400 words, due before each session.
Class participation and professionalism	Students were expected to fully participate in class discussions and complete individual and team activities. Each student was expected to adhere to a learning environment that was engaging and supportive.
Personal Health Improvement Plan (PHIP)	While completing readings and participating in discussions on health challenges, students were expected to work independently to identify a personal health priority and establish a plan for improvement. The final project included a written reflection and a 10-minute in-class presentation.
Culture of Wellbeing Team Project Presentation	Students collaborated within a small group of peers to advance community wellbeing. Using improvement process tools and evidence-based wellbeing strategies, teams planned and proposed a Culture of Wellbeing initiative that could be activated within the school community. The final project included a 10-minute presentation.

Table S2: Pre-post survey given to students immediately before and immediately after the “Culture of Wellness” course

Assessments	Survey Main Question	Response: Strategies / Questions	Scale
**Part 1: Skills Assessment**	*“To what degree are you confident in your ability to apply the following strategies to improve wellbeing?”*	**Strategies provided:**<br>Mindfulness; self reflection, resilience, narrative writing, healthy eating, exercise, relationships, time management, leading change, emotional intelligence, prioritizing purpose, cognitive reframing, appreciative inquiry	1. Not at all<br>2. Minimally<br>3. Somewhat<br>4. Mostly<br>5. Completely
**Part 2: Wellbeing Assessment**			
Quality of life	*“Please select the number that best describes your feelings during the past week, including today. How would you describe:”*	*“Your overall quality of life?”*<br>*“Your overall mental (intellectual) wellbeing?”*<br>*“Your overall physical wellbeing?”*<br>*“Your overall emotional wellbeing?”*<br>*“Your level of social activity?”*<br>*“Your spiritual wellbeing?”*	Range from 1-10:<br>(1 = As bad as it can be; 10 = As good as it can be)
Perceived stress	*“In the last month, how often have you felt:”*	*“That you were unable to control the important things in life?”*<br>*“Confident about your ability to handle your personal problems?”*<br>*“That things were going your way?”*<br>*“Difficulties were piling up so high that you could not overcome them?”*	1. Never<br>2. Almost never<br>3. Sometimes<br>4. Fairly often<br>5. Very often
Burnout	*“Over the last month:”*	*“Have you felt burned out from your work?”*<br>*“To what degree have you felt burned out?”*	Yes or No<br><br>1. Not at all<br>2. Minimally<br>3. Somewhat<br>4. Mostly<br>5. Completely

Mindfulness	*"Over the last month:"*	*"To what degree have you felt present in the moment?"*	1. Not at all<br>2. Minimally<br>3. Somewhat<br>4. Mostly<br>5. Completely

Table S3: Personal Health Improvement Plan topics chosen by students in the 2020 course

PHIP Topic	Number of Students
Exercise	6
Meditation	4
Journaling/Writing/Reading	3
Sleep hygiene	3
Getting outside/enjoying nature	2
“Unplugging” from technology	2
Time management	1
Increasing water consumption	1

Table S4: Perceived Stress Scale pre-course survey results

**Perceived Stress Scale Question (**In the last month, how often have you felt…)[1]	**2019 mean (n=54)**	**2020 mean (n=19)**	**Percent Change**	**p-value**
That you were unable to control the important things in life?	3.01	3.05	1.33	0.12
Confident about your ability to handle your personal problems?	3.82	4.05	6.02	0.15
That things were going your way?	3.11	3.32	6.75	0.15
Difficulties were piling up so high that you could not overcome them?	3.48	2.68	-22.99	0.17

[1]The scale ranges from “Never,” assigned a score of 1, to “Very often,” assigned a score of 5

Table S5: SMART goals for each of the 6 team projects

Teams	SMART Goal
Team 1	For the duration of Winter Term, students will schedule 30 mins of outdoor walking. They will gauge success by having completed the goal and then self-assessing their stress using the Perceived Stress Scale.
Team 2	All students will journal using a self-reflection prompt for 10 minutes at the end of the last class of the day.
Team 3	Participating students will consume the recommended amount of water daily, 0.75 ounces of water per pound of body weight, for one month.
Team 4	All students spend 15 cumulative minutes per day outside without any technology for the next 30 days.
Team 5	Students will participate in an optional five-minute mindfulness meditation prior to the start of our first morning lecture.
Team 6	Students will free up 90 min of time each day for whatever they need by keeping a detailed time log and using the “R Principles” to reorganize life.

Figure S1: Perceived stress pre-course survey results in 2019 versus 2020

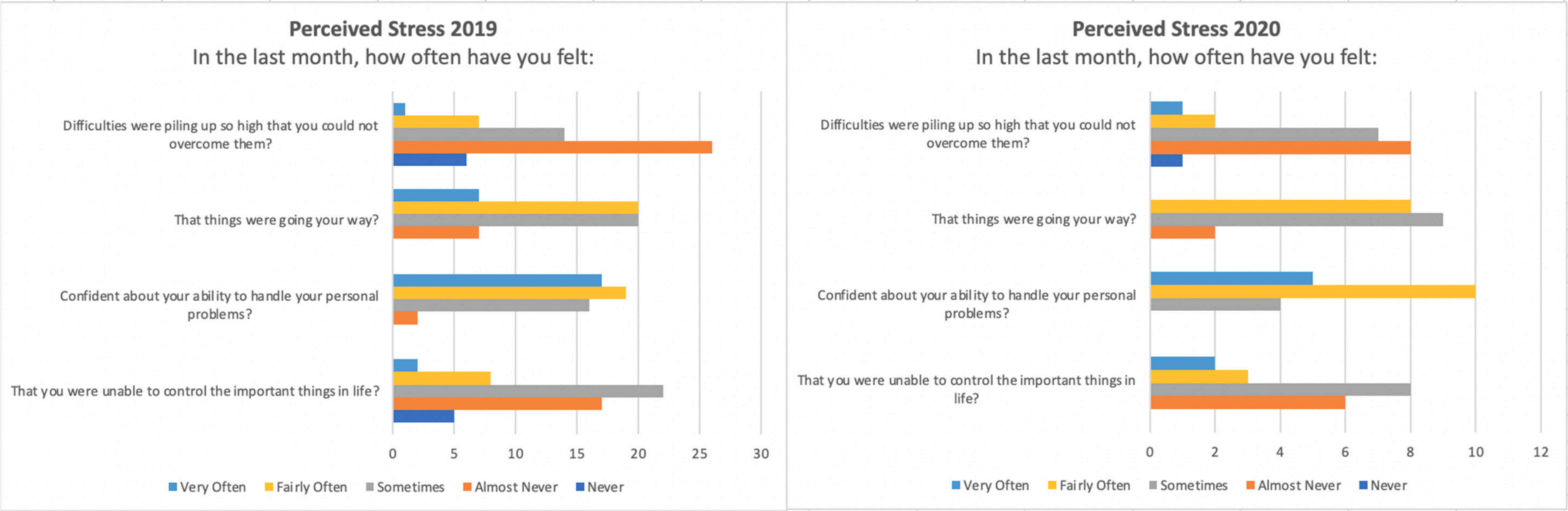

Figure S2: Five-month post-course survey question on importance of strategies to the students' personal wellness

How important are the wellbeing strategies learned in PH104 to your personal wellness currently?

14 responses

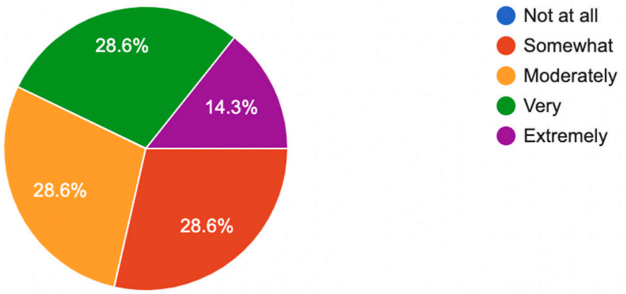

Figure S3: Five-month post-course survey on confidence in ability to apply the strategies

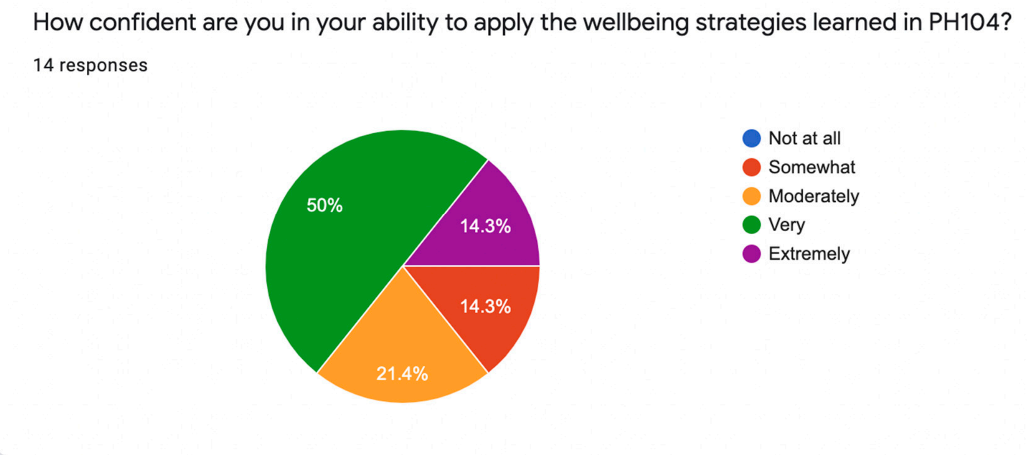

Figure S4: Five-month post-course survey question on frequency of application of strategies

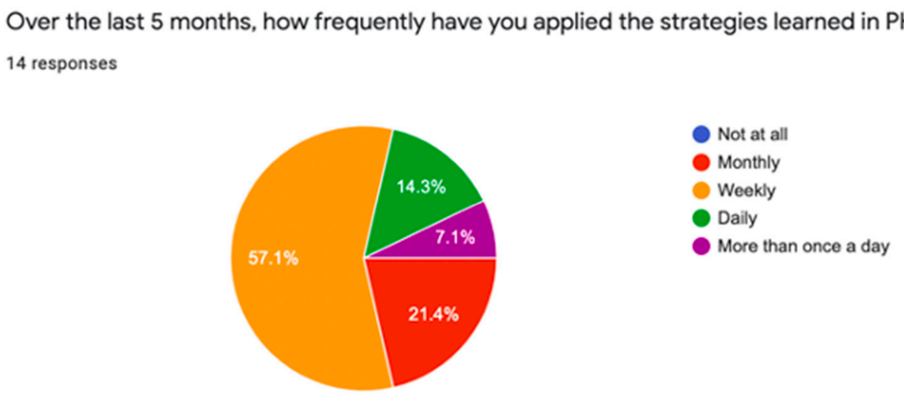

Supplement: Supplementary file 1 [file ijerph-21-00590-s001.zip › ijerph-2923295-supplementary.pdf]
